# The Use of Antibody-Antibiotic Conjugates to Fight Bacterial Infections

**DOI:** 10.3389/fmicb.2022.835677

**Published:** 2022-03-07

**Authors:** Marco Cavaco, Miguel A. R. B. Castanho, Vera Neves

**Affiliations:** Faculdade de Medicina, Instituto de Medicina Molecular João Lobo Antunes, Universidade de Lisboa, Lisbon, Portugal

**Keywords:** antibody, antibiotic, antibody-antibiotic conjugates, bacteria, infections, resistance

## Abstract

The emergence of antimicrobial resistance (AMR) is rapidly increasing and it is one of the significant twenty-first century’s healthcare challenges. Unfortunately, the development of effective antimicrobial agents is a much slower and complex process compared to the spread of AMR. Consequently, the current options in the treatment of AMR are limited. One of the main alternatives to conventional antibiotics is the use of antibody-antibiotic conjugates (AACs). These innovative bioengineered agents take advantage of the selectivity, favorable pharmacokinetic (PK), and safety of antibodies, allowing the administration of more potent antibiotics with less off-target effects. Although AACs’ development is challenging due to the complexity of the three components, namely, the antibody, the antibiotic, and the linker, some successful examples are currently under clinical studies.

## Introduction

Worldwide, life expectancy has increased significantly over the last century. During the same period, the mortality associated with infectious diseases has declined due to significant improvements in sanitation, nutrition, vaccination, medical practices, the discovery of effective drugs, and the creation of robust and responsive healthcare systems in many countries (Foreman et al., 2018). Nevertheless, infectious diseases remain a global health threat. Some infectious diseases are endemic to many areas, causing significant and steady burdens. Others are globally spread, causing the death of millions of people (Baker et al., 2021). In addition, the recurrence of emerging infections with the capacity for rapid expansion remains an important and acute threat for human beings (Collignon et al., 2018; Bloom and Cadarette, 2019).

To worsen matters, many drugs that have contributed to decreasing the mortality rates associated with numerous infectious diseases are declining in efficacy (Naylor et al., 2018). The rising of antimicrobial resistance (AMR) is one of the biggest threats of twenty-first-century medicine and the leading cause for therapeutic failure in the field of infectious diseases (Bloom and Cadarette, 2019). Most of the AMR are related to bacteria, and the infections are typically nosocomial (i.e., occurs in a hospital or other health care facility). Unlike pandemic threats, resistant pathogens’ proliferation rate is slow; however, they have expanded worldwide (Bloom and Cadarette, 2019). Moreover, there are only a limited number of effective treatments for some resistant pathogens (Leekha et al., 2011; Pérez-Rodríguez and Taban, 2019). Consequently, the development of drugs that are effective and safe is urgent. Unfortunately, the development of new drugs is a slow process (Ventola, 2015; World Health Organization, 2018). In addition, the pharmaceutical industry’s disregard for new antibacterial agents can be linked to the absence of economic incentives and challenging regulatory requirements, hindering the development of new therapeutic agents in this field (Aslam et al., 2018).

Numerous organizations, such as the Centers for Disease Control and Prevention (CDC) and the World Health Organization (WHO), have declared AMR to be a “global public health concern” (Michael et al., 2014; Spellberg et al., 2016). The CDC and the WHO released a priority pathogens list for research and development of new anti-infective agents, but the situation keeps exacerbating (Table 1; Ventola, 2015). Predictably, it will lead to 10 million people dying every year and a 2–3.5% reduction in Gross Domestic Product (GDP) by 2050. However, these values might be underestimated since these studies (1) looked only at a subset of drug-resistant bacteria and public health issues; and (2) only GDP was considered a financial metric. Other problematics, like the social and healthcare costs, were excluded (O’Neil, 2014).

**TABLE 1:** WHO and CDC priority bacteria list for R&D.

Priority	WHO^a^	CDC^b^
Critical	*Acinetobacter baumannii* (carbapenem-resistant) *Pseudomonas aeruginosa* (carbapenem-resistant) *Enterobacteriaceae*^c^ (carbapenem-resistant; 3rd generation cephalosporin-resistant)	*Acinetobacter baumannii* (carbapenem-resistant) *Clostridioides difficile* *Enterobacteriaceae* (carbapenem-resistant) *Neisseria gonorrhoeae* (drug-resistant)
High	*Enterococcus faecium* (vancomycin-resistant) *Staphylococcus aureus* (methicillin-resistant; vancomycin intermediate and resistant) *Helicobacter pylori* (clarithromycin-resistant) *Campylobacter* (fluoroquinolone-resistant) *Salmonella* spp. (fluoroquinolone-resistant) *Neisseria gonorrhoeae* (3rd generation cephalosporin-resistant; fluoroquinolone-resistant)	*Campylobacter* (drug-resistant) *Enterobacteriaceae* (ESBL-producing) *Enterococci* (vancomycin-resistant) *Pseudomonas aeruginosa* (multidrug-resistant) *Salmonella spp.* (drug-resistant) *Shigella* spp. (drug-resistant) *Staphylococcus aureus* (methicillin-resistant) *Streptococcus pneumoniae* (drug-resistant) *Mycobacterium tuberculosis* (drug-resistant)
Medium	*Streptococcus pneumoniae* (penicillin-non-susceptible) *Haemophilus influenzae* (ampicillin-resistant) *Shigella* spp. (fluoroquinolone-resistant)	Streptococcus (erythromycin-resistant; clindamycin-resistant)

*^a^World Health Organization. ^b^Centers for Disease Control and Prevention. ^c^Enterobacteriaceae include: Klebsiella pneumonia, Escherichia coli, Enterobacter spp., Serratia spp., Proteus spp., and Providencia spp., Morganella spp.*

**FIGURE 1:**
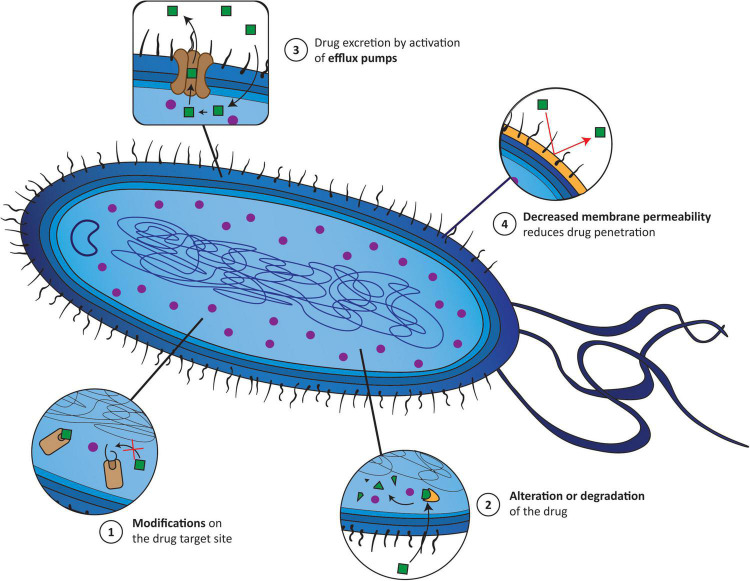
The main mechanisms responsible for the appearance of antimicrobial resistance (AMR) correspond to (1) modifications of the target site of antibiotics; (2) alterations or even degradation of the antibiotic; (3) antibiotic efflux *via* efflux transporters; and (4) reduced antibiotic penetration into bacteria through decreased membrane permeability. *Green squares* (1–4), *drug incapable of accumulating inside the bacteria; purple circle* (1–4), *drug accumulating inside bacteria; yellow cone* (2), *enzyme; yellow wall* (4), *bacterial wall.*

Understanding the various mechanisms of AMR is the key to addressing this issue properly. The main molecular mechanisms by which, for instance, bacteria become resistant to an antibiotic can be divided into: (1) modification of the target site of antibiotics; (2) alteration or even degradation of the antibiotic; (3) antibiotic efflux via efflux transporters; and (4) reduced antibiotic penetration into bacteria through decreased membrane permeability (Munita and Arias, 2016; Reygaert, 2018). These mechanisms can be present alone or coexist in bacteria (Figure 1). Therefore, alternative treatment strategies have to be designed to overcome AMR and increase the effectiveness of antibiotics. One of the most explored methodologies is antibiotic resistance breakers (ARBs) (Gill et al., 2015; Laws et al., 2019), which can be combined with antibiotics. The major ARB classes under investigation include modifying-enzyme inhibitors (e.g., β-lactamase inhibitors, aminoglycoside-modifying enzyme inhibitors), membrane permeabilizers (e.g., polymyxins, antimicrobial peptides, plant-derived phenolic compounds), and efflux pump inhibitors (e.g., catechin gallates, alkaloids, peptidomimetics). The use of ARBs is extremely attractive since, in theory, it reduces the antibiotic selection pressure, which can slow the onset of resistance, and because it alleviates the side effects of some antibiotics by widening the therapeutic window (Laws et al., 2019).

Another interesting approach looks beyond small-molecule drugs to biologics and related technologies. The use of nanotechnology is emerging in different areas of medicine as an attractive therapeutic approach (Wang et al., 2017; Kumar et al., 2018). In the particular case of infectious diseases, nanoparticles can be coupled with antimicrobial agents to improve physicochemical properties, or directly target bacteria, causing their elimination. Antimicrobial peptides are also getting more attention because of the low frequency of adverse events, broad activity spectrum, and innovative mechanism of action, which comprises a direct action toward bacterial membranes and/or unspecific targeting of proteins, DNA, RNA, and regulatory enzymes (Zharkova et al., 2019; Luong et al., 2020; Moretta et al., 2021).

However, one of the most promising strategies is targeted delivery to overcome resistance while reducing the selection pressure of antibiotics, through antibody-based strategies, such as antibody-drug conjugates (ADCs). Numerous ADCs are being used in clinical practice, or evaluated in clinical trials, especially in cancer therapy (Chau et al., 2019). Based on the knowledge acquired in the development of ADCs, researchers engineered antibody-antibiotic conjugates (AACs) using bacteria-specific antibodies (Mariathasan and Tan, 2017).

## The Use of Antibacterial Monoclonal Antibodies

Amil von Behring and Shibasaburo introduced the use of antibodies, in the form of serum, to treat infectious diseases in 1890 (Yamada, 2011). Although successful toward numerous pathogens, such as *Corynebacterium diphtheria, Streptococcus pneumonia, Neisseria meningitides, Haemophilus influenzae, Group A Streptococcus, and Clostridium tetani*, the allergic reactions, heterogeneity between lots, and limited spectrum, led to its replacement in the 1930s by simpler antibiotics (Reichert and Dewitz, 2006). Overall, most antibiotics are easy to manufacture, more easily formulated, safe, and very effective. Thus, they have become dominant over the last 80 years (Chan et al., 2009).

In 1975 the discovery of hybridoma technology and recent advances in monoclonal antibody (mAb) engineering renewed the interest in developing antibacterial mAbs (Schroff et al., 1985; Köhler and Milstein, 2005). mAbs are widely used to treat immune deficiencies, cancers, multiple sclerosis, rheumatoid arthritis, and psoriasis. Concerning bacterial infections, numerous antibodies are under clinical evaluation. Yet, only three antibodies are approved for use in treating bacterial infections (Table 2; Motley and Fries, 2017).

**TABLE 2:** Monoclonal antibodies and antibody-based biologics that have been tested in clinical trials for use in bacterial infections.

Antibody	Company	Species	Isotype	Pathogen (target)	Mechanism of action	Indication	Phase
514G3	XBiotech	Human	IgG3	*Staphylococcus aureus* (Protein A—SpA)	Opsonophagocytosis	*Staphylococcus aureus* bacteremia	Phase I/II
Aerucin	Aridis	Human	IgG1	*Pseudomonas aeruginosa* (alginate)	Opsonophagocytosis; complement-mediated bacterial killing	Pneumonia	Phase II
ASN100 - ASN-1 and ASN-2 mix	Arsanis	Human	IgG1(κ)	*Staphylococcus aureus* (α-hemolysin—HIa, HIgAB, HIgCB, LukED, LukSF, and LukGH)	Toxin neutralization	Pneumonia prevention	Phase II
Bezlotoxumab (ZINPLAVA^®^) - MK-6072 - CDB-1 - MDX-1388	Merck & Co.	Human	IgG1(κ)	*Clostridioides difficile* (Enterotoxin B)	Toxin neutralization	Prevention of *Clostridioides difficile* infection recurrence	Approved
DSTA4637S	Genentech	Human	IgG1	*Staphylococcus aureus* (β-*O*-linked *N*-acetylglucosamine on wall teichoic acids—WTA)	Antibody-antibiotic conjugate	Pneumonia	Phase I
MEDI-3902 - biS4aPA	MedImmune	Human bispecific	IgG1(κ)	*Pseudomonas aeruginosa* (PsI and PerV)	Opsonophagocytosis; complement-mediated bacterial killing	Pneumonia	Phase II
Suvratoxumab - MEDI-4893	Astra Zeneca	Human	IgG1(κ)	*Staphylococcus aureus* (α-hemolysin—HIa)	Toxic neutralization	Pneumonia	Phase II
NTM-1632	NIAID	Humanized	IgG1	*Clostridium botulinum* (Botulinum neurotoxin B)	Toxin neutralization	Botulism	Phase I
Obiltoxaximab (ANTHIM^®^) - ETI-204	Elusys	Mouse/Human chimeric	IgG1(κ)	*Bacillus anthracis* (Protective antigen—PA)	Toxin neutralization	Inhalation anthrax	Approved
Pagibaximab - BSYX-A110	Biosynexus	Mouse/Human chimeric	IgG	*Staphylococcus epidermidis* (Lipoteichoic acid—LTA)	Opsonophagocytosis; complement-mediated bacterial killing	Septicemia	Phase II
Panobacumab (Aerumab) - AR-101 - KBPA-101	Aridis	Human	IgM (κ)	*Pseudomonas aeruginosa* (LPS O-antigen—O11)	Opsonophagocytosis; complement-mediated bacterial killing	Pneumonia	Phase II/III
Pritoxaximab	Bellus Pharmaceuticals	Mouse/Human chimeric	IgG1(κ)	*Escherichia coli* (Shiga toxin type 1, and Shiga-like toxin 1)	Toxin neutralization	STEC^a^ infection causing diarrhea and HUS^b^	Phase II
Raxibacumab (ABthrax^®^)	GlaxoSmith Kline	Human	IgG1(λ)	*Bacillus anthracis* (Protective antigen—PA)	Toxin neutralization	Inhalation anthrax	Approved
SAR279356 - F598	Sanofi	Human	IgG1	Multiple pathogens (Poly-*N*-acetylglucosamine)		Prevention of bacterial infections	Phase II
Setoxaximab	Bellus Pharmaceuticals	Mouse/Human chimeric	IgG1(κ)	*Escherichia coli* (Shiga toxin type 2, and Shiga-like toxin 2)	Toxin neutralization	STEC infection causing diarrhea and HUS	Phase II
Tosatoxumab (Salvecin) - AR-301	Aridis	Human	IgG1	*Staphylococcus aureus* (α-hemolysin—HIa)	Toxin neutralization	Inhalation anthrax	Phase II

*^a^STEC, Shiga-like toxin-producing Escherichia coli. ^b^HUS, Hemolytic-uremic syndrome. clinicaltrials.gov.*

The main mechanisms of action of mAbs are distinct from conventional small-molecule antibiotics and are less prone to drug resistance. They can be divided into (1) anti-virulence mechanisms; and (2) bactericidal mechanisms. The blockage of bacterial virulence mechanisms limits collateral damage, such as the development of drug resistance and helps both the host’s innate and adaptive immune defense mechanisms. The most effective approach has been toxin neutralization (Sawada-Hirai et al., 2004). Numerous pathogenic bacteria (*Corynebacterium diphtheriae, Bordetella pertussis, Vibrio cholerae, Bacillus anthracis, Clostridium botulinum, Clostridium tetani, Clostridioides difficile*, and *enterohaemorrhagic Escherichia coli*) cause disease by releasing toxins. The administration of mAbs that binds to soluble toxins and form antibody-toxin complexes leading to clearance by the reticuloendothelial system. More recently, other virulence factors have been assessed, such as the type III secretion system, adhesins and pili, and outer membrane transporters. Unlike exotoxins, these antigens are exposed on the bacterial membrane so, in addition to neutralization, antibodies targeting these antigens can also trigger bactericidal effects (Nagy et al., 2017).

Ideally, targeted antigens must be abundant and freely exposed to allow a proper binding by antibodies, and limited to bacteria to avoid off-target effects. In most cases, mAbs cannot elicit direct bacteria killing. They depend on the co-operation of phagocytic cells (antibody-dependent cellular cytotoxicity—ADCC), and/or complement (complement-dependent cytotoxicity—CDC) (Lu et al., 2018). Classical ADCC involves antibody binding to bacteria, followed by the recruitment of professional phagocytes (i.e., monocytes/macrophages, neutrophils, and dendritic cells) *via* fragment crystallizable (Fc)- gamma receptors (FcγRs), which results in the release of perforin and/or granzyme that drives cell death (Weiner, 2015). CDC is another important bactericidal mechanism that involves the binding of mAbs on the bacterial surface that enhances the recruitment and binding of soluble complement factors, including C1q, to the Fc domain of the mAb. This binding leads to the activation of the complement cascade, and the formation of the membrane attack complex (Melis et al., 2015).

The antibacterial mechanism of action of the approved mAbs consists in the neutralization of exotoxins from Gram-positive pathogens (Wang-Lin and Balthasar, 2018). Raxibacumab is a human mAb that targets the protective antigen (PA) component of the toxin of *Bacillus anthracis*. It is approved for use in combination with appropriate antibacterial drugs and for prophylaxis of inhalational anthrax. Obiltoxaximab is also an anthrax toxin neutralizing mAb targeting PA. It has the same indication and function as raxibacumab. The other mAb is bezlotoxumab, which is a human IgG1 that reduces the recurrence of *Clostridioides difficile* infection. Nine others are under development; five against *Staphylococcus aureus*, three targeting *Pseudomonas aeruginosa*, and one for *Escherichia coli* (Table 2).

The major advantages of mAbs are the optimal selection of the antibody target and their high specificity, which allows less off-target effects and less selective pressure for cross-resistance. Nevertheless, there are still some challenges concerning their cost production and systemic administration (Bebbington and Yarranton, 2008; McConnell, 2019).

## Antibody-Antibiotic Conjugates as an Alternative

To date, more than 100 ADCs are under evaluation worldwide, mainly in the treatment of cancer (Zhao et al., 2020; Pettinato, 2021). Although this antibody-based molecular platform is relatively simple, the development of ADCs is challenging. Early ADCs demonstrated high immunogenicity, low potency, and suboptimal target selectivity, since mouse domains were used to engineer antibodies (Beck et al., 2017). Nevertheless, recent humanization strategies contribute to developing more effective next-generation ADCs, which are becoming more potent, more selective, and less immunogenic. The lessons learned with ADCs for cancer have been translated into infectious diseases. Naturally, instead of a cytostatic drug, highly potent antibiotics are conjugated to mAbs, generating AACs (Mariathasan and Tan, 2017).

### Antibody-Antibiotic Conjugate Domains

**FIGURE 2:**
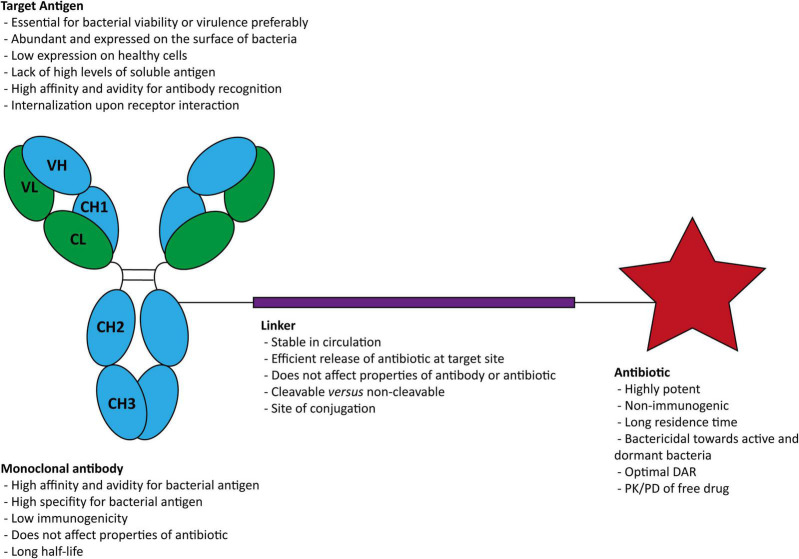
Structure of an antibody-antibiotic conjugate and general characteristics of (i) the target antigen, (ii) the antibody, (iii) the linker, and (iv) the antibiotic. DAR, drug-antibody ratios; PK, pharmacokinetic; PD, pharmacodynamic.

The components necessary in an AAC for infectious diseases are a bacterial antigen-specific mAb, a stable cleavable or non-cleavable chemical linker, and a potent antibiotic (Figure 2).

#### Antibody

The primary function of the mAb is to selectively target and deliver the antibiotic directly into the infectious site (Maxson and Mitchell, 2016). Thus, selecting the target antigen to which the AAC binds is essential. The ideal target antigen must be homogeneously expressed at the surface of targeted bacteria, and almost absent on healthy tissues to limit off-target effects (Staudacher and Brown, 2017). The mAb should bind with high affinity, to allow selective accumulation and durable retention. In addition, antibody binding must result in the internalization of the antibody-antigen complex to enable the antibiotic’s intracellular delivery (Chari et al., 2014).

Carbohydrates are considered a potential target for antibodies owing to their high abundance and significance in bacterial pathogenesis (Soliman et al., 2020). One of the classes within the carbohydrate family is the lipopolysaccharides (LPS) that consists of a common core saccharide proximal to the lipid and an O-antigen (O-Ag) that differs between species and strains of bacteria (Cross, 2014). Another class corresponds to capsular polysaccharides (CPS), which are generally long repeating saccharide structures that surround and protect many bacteria and contribute to cellular adhesion. Targeting some components within this class has been one of the most successful strategies (Lang et al., 1991). In addition, other targets, such as highly conserved exopolysaccharides, pilus formation proteins, and extracellular vesicle components, which are essential for bacteria pathogenesis, have also been studied as immunotherapy targets (Motley and Fries, 2017).

Along with the importance of the target selection, attention must be given to selecting the IgG type of immunoglobulin (e.g., IgG1, IgG2, IgG3, and IgG4), since they can impact various effector functions of the AAC (Vidarsson et al., 2014). The most commonly used IgG isotype is the IgG1. It can strongly induce ADCC and CDC, and has one of the longest half-lives known (Beck et al., 2017). Although AACs do not require the antibody itself to possess any additional activity in addition to binding, these features may confer additional therapeutic benefit (Mariathasan and Tan, 2017).

#### Antibiotic Payload

Numerous antibiotics have been investigated for the treatment of infectious diseases. Nevertheless, their clinical application is sometimes hampered by the bioavailability, toxicity, and biodistribution (York, 2020). These drawbacks and their short half-life and short-acting effect lead to therapeutic regimens where more and high doses are required. The conjugation to mAbs is an elegant solution to overcome these limitations (Cavaco et al., 2018). Antibiotics are the main effector of the AACs. Among other important features, these antibiotics 1) should have bactericidal potency in the subnanomolar range; 2) the antibiotic must contain a functional group for conjugation to mAbs; and 3) the antibiotic should be soluble and stable under physiological conditions (Cavaco et al., 2018).

So far, the use of rifamycin-type antibiotics has been the most successful class conjugated to mAbs (Mariathasan and Tan, 2017). However, different antibiotic moieties selected from clindamycin, novobiocin, retapamulin, daptomycin, GSK-2140944, CG-400549, sitafloxacin, teicoplanin, triclosan, naphthyridine, radezolid, doxorubicin, ampicillin, vancomycin, imipenem, doripenem, gemcitabine, dalbavancin, and azithromycin were also successfully conjugated to mAbs and are under investigation to fight infectious diseases (Cal et al., 2017).

#### Linker

An essential structural component of an AAC is the engineering of a linker responsible for the connection between cargo and payload, such as an antibiotic and antibody. On the one hand, the linker must be stable in blood circulation to keep the antibiotic attached to the antibody (Chau et al., 2019). Premature release of antibiotics in circulation can decrease AAC efficiency and increased AAC toxicity (Donaghy, 2016; Buecheler et al., 2018). On the other hand, it must retain the ability to release the payload once the antibody is internalized. Another property to be considered is the linker hydrophobicity (Su and Zhang, 2021). The connection of a hydrophobic linker and a hydrophobic payload might promote aggregation, which compromises the AAC stability. Different strategies have been widely applied to improve their physicochemical properties due to its usefulness. Currently, there are two major classes: cleavable linkers and non-cleavable linkers (Tsuchikama and An, 2018).

Most ADCs in clinical trials or in preclinical studies are composed of cleavable linkers. These linkers include motifs that are sensitive to physiological stimuli, such as low pH or proteolytic cleavage, to release the drug from the antibiotic-carrier (Dan et al., 2018). This system allows researchers to estimate the potency of unconjugated payload based on known pharmacokinetic (PK)/pharmacodynamic (PD) parameters of the free payload.

Hydrazone linker is acid-cleavable, which allows the conjugate to remain stable in the circulation at the neutral pH (Tsuchikama and An, 2018). Nevertheless, it releases free drug through hydrolysis in the acidic cellular compartment, in either the acidic endosomes (pH 5.0–6.0) or the lysosomes (pH about 4.8). So far, the ADCs engineered with these linkers have been associated with the non-specific release of the drug in clinical trials since hydrazone linker undergo slow hydrolysis under physiological conditions (pH 7.4, 37°C). Another important linker, which is used in the AAC under clinical evaluation, is the cathepsin B-responsive linker (Lehar et al., 2015). Cathepsin B is a lysosomal protease that is overexpressed in numerous cancer cells and bacterial infections (Gondi and Rao, 2013). It cleaves preferentially certain sequences, such as phenylalanine-lysine (Phe-Lys) and valine-citrulline (Val-Cit). Upon internalization of the AAC, the antibiotic is released in the lysosomes in a traceless manner. This linker has been one of the most successful cleavable linkers for ADCs. Another promising cleavable linker is the glutathione sensitive linker. This strategy takes advantage of the higher concentration ratio of glutathione between the cytoplasm and the extracellular environment (Mills and Lang, 1996). In circulation, the disulfide bond is highly stable. However, upon internalization, the presence of high amounts of glutathione cleaves the disulfide bond and releases the free payload (Saito et al., 2003). Pyrophosphate diester linker has demonstrated a higher aqueous solubility and circulatory stability than traditional linkers. Upon internalization, the pyrophosphate diester gets quickly cleaved the linker through the endosomal-lysosomal pathway to liberate the payload (Kern et al., 2016). Finally, quaternary ammonium salt linker is also applied. This strategy was designed to take advantage of a novel connection to tertiary amines (Pillow, 2017). These tertiary amines are commonly found in numerous anticancer drugs and antibiotics (Dan et al., 2018; Kostova et al., 2021). So far, the approach relies on the removal of a methyl group to connect the linker, which might affect the stability and efficacy of the drug. This new strategy allowed developing potent ADCs and AACs, with increased stability of the conjugates.

Non-cleavable linkers, often containing a thioether bond, are resistant to proteolytic degradation, ensuring greater stability than of cleavable linkers. They rely on the lysosomal degradation to release the payload upon internalization (Birrer et al., 2019). The effectiveness of this linker depends on the stability of the payload. The drug must maintain its activity, despite the connection to the linker (Su et al., 2021). These ADCs are considered to have improved therapeutic index, owing to improved plasma stability (Ponziani et al., 2020).

#### Site-Specific Conjugation

Selection of the antibody, antibiotic, and linker is critical to ensure the engineered AAC that is efficient and non-toxic (Leung et al., 2020). Another important feature to be considered in the selection of the strategy applied in the conjugation between the antibiotic to the antibody. Chemical conjugation and enzymatic conjugation are the two most important methods currently in use (Tsuchikama and An, 2018).

There are numerous chemical conjugation methods that researchers can use to conjugate an antibiotic to an antibody (Yao et al., 2016). The basic principle consists in a controlled reaction between accessible amino acid residues on the surface of the antibody and reaction handle installed on the linker. Depending on the method selected, a mixture of AAC species with variable drug-antibody ratios (DARs) and tethering sites is obtained (Tsuchikama and An, 2018). This heterogeneity may compromises the efficiency, safety, and stability of the AAC. Thus, the selection of the most appropriate method for each complex system is important.

The most common conjugation methods is lysine amide coupling. Lysines are usually exposed on the surfaces of the antibodies, being accessible for conjugation to an activated carboxylic acid group. However, antibodies contain about 80 lysine amino acid residues, resulting in high heterogeneity (Mueller et al., 1988). This heterogeneity relates to both different DARs and conjugation sites. The former can be minimized by adjusting the stoichiometry of the drug and antibody used in the reaction. The latter requires the blocking of selected reactive groups. In addition, the antibody-binding affinity might also be compromised due to the importance of some lysine amino acid residues in the antibody-antigen interaction (Chari, 2008). Overall, the lysine-based method requires fine tuning optimization to overcome the high heterogeneity.

Cysteine coupling is an alternative; this methodology requires a reaction between cysteine amino acid residues of the antibody and a thiol-reactive group present on the antibiotic to form a disulfide bond (Cao et al., 2019; You et al., 2021). Nevertheless, there are no free thiols on antibodies, as virtually all cysteine amino acid residues form disulfide bonds due to their high reactivity. For instance, in IgG1, the most common IgG subtype used in the engineering of ADCs, there are 4 interchain and 12 intrachain disulfide bonds (Tsuchikama and An, 2018). The former are not essential for the stability of the antibody. Thus, they can be reduced under mild condition, creating 2, 4, 6, or 8 free thiols. Generally, the 12 intrachain disulfides remain intact. This strategy is considered superior to the lysine amide coupling, due to the engineering of more homogenous ADCs, as the number of conjugation sites is limited. An improvement to this approach is the introduction of two new cysteine amino acid residues for selective antibody attachment (Junutula et al., 2008). This engineered cysteine technology, called THIOMAB™, enables the creation of very homogenous AACs with a DAR of 2. Another interesting strategy is the disulfide re-bridging. Theoretically, this strategy creates site-specific conjugation points that allow structural stability, homogeneity, and DARs of 4 (Behrens et al., 2015). The insertion of entire domains or proteins into antibodies also enables the generation of homogenous conjugates. The main method in this category is the expressed protein ligation (EPL), which relies on a self-splicing intein to activate the C-terminal of the target protein and thus formed a new amide bond with the drug payload (Kline et al., 2015).

Finally, non-natural amino-acid engineering is getting more attention. The introduction of non-natural amino acid residues in specific points of the antibody to strictly control DARs is a promising strategy (Kline et al., 2015). Researchers have used the following non-natural amino acid residues: acetylphenylalanine (Tian et al., 2014), p-azidomethyl-L-phenylalanine (Zimmerman et al., 2014), and N6-((2-azidoethoxy)carbonyl)-L-lysine (VanBrunt et al., 2015). Although the use of non-natural amino acid residues allows a high homogeneity, this methodology requires special techniques and biological agents for the genetic engineering process that can trigger undesired immunological response (Tsuchikama and An, 2018).

The use of several enzymes has been proposed for conjugating drugs to antibodies. The specificity of these enzymes modifies the antibody in a site- or amino acid sequence-specific manner. Thus, enzymatic approaches generally allow for site-specific conjugation leading to tightly controlled DARs (Beck et al., 2017).

There are three main enzyme-dependent conjugation methods. The first is the transpeptidation using sortase. Sortase A is an enzyme from *Staphylococcus aureus* that recognizes a Leu-Pro-x-Thr-Gly (LPXTG; X: any amino acid residue) motif (Zhang et al., 2004). This enzyme cleaves the Thr-Gly bond and attaches an olygoglycine-containing molecule. In the literature, it is possible to find numerous successful examples of the conjugation of peptides, proteins, nucleic acids, and other molecules (Beerli et al., 2015). The second methodology implies the use of transpeptidation using microbial transglutaminase. The use of transglutaminases has been successfully applied in the site-specific incorporation of drug payloads into antibodies (Witte et al., 2012). These enzymes catalyze transpeptidation, where a primary amine-containing linker is covalently conjugated to the primary amine side chain of a specific Glu (Q295) in antibodies. The resulting product has a defined DAR of 2. The absence of genetic engineering is advantageous over the other conjugation methods (Dennler et al., 2014). Finally, the N-Glycan engineering. All the IgG classes have a conserved Asn (N297) in the Fc domain and the N-glycan on this residue. Thus, this site-specific point is very attractive to make homogenous conjugations. The incorporation of an aldehyde group on the N-glycan terminus is one of the approaches that researchers use (Zhou et al., 2014). These groups can then be conjugated to aminooxy-functionalized drug payloads. Nevertheless, some heterogeneity might be observed. Another approach relies on the incorporation of non-natural saccharides possessing orthogonal reaction handle into the antibody. The most significant advantage of this approach consists of the reproducibility within conjugations.

These different components must be selected carefully since they can affect the overall efficacy of AACs. In addition, the PK and PD of the conjugates must be considered since each component is essential and can contribute differently to the efficacy of the conjugate. Thus, a proper PK/PD evaluation is vital in the validation of a specific AAC.

### Key Factors Affecting Absorption, Distribution, Metabolization, and Excretion

The use of AACs toward infectious diseases has increased popularity in the last decade but the complexity of AACs introduced new challenges related to stability, catabolism, and elimination, which are the main factors affecting the absorption, distribution, metabolization, and excretion (ADME) of antibody-based therapies. Understanding these parameters is essential to increase the efficacy and to reduce the toxicity of these agents (Tibbitts et al., 2016). The major challenge with AACs is their narrow therapeutic index; thus a proper characterization of their PK/PD properties is mandatory.

#### Pharmacokinetic Considerations

As mentioned before, an AAC is composed of an antibody, an antibiotic, and a linker. The antibody and the antibiotic are mainly responsible for the efficacy of the AAC, whereas the linker is extremely important for the stability of the system. The entire AAC and specific components must be studied individually and together for a proper ADME characterization, which are important to describe the behavior of a therapeutic drug within an organism (Lucas et al., 2018).

The oral route is the primary choice for drug administration since it allows better patient compliance and a lower cost of therapy. Nevertheless, the oral route poses a challenge for therapeutic proteins, which cause degradation of biopharmaceutics in the gastrointestinal tract (Ibrahim et al., 2020). As a result, antibody-based therapies are usually administrated either intravenously (iv) or subcutaneously (sc). The majority of mAbs for oncology are administrated by an iv infusion with a 100% bioavailability; whereas for inflammatory diseases, mAbs are usually administrated via sc injection with a 50–80% bioavailability (Zhao et al., 2013). Although the sc route is preferable over the iv route for most interventions, for ADCs and AACs, the sc administration is discouraged, due to potential reactions of the payload and off-target effects mediated by immune cells in the skin (Lucas et al., 2018).

The next step following administration is the distribution of the antibody complex in the organism. The presence of the antibiotic in AACs relative to mAbs might not only affect the distribution of AACs, but also interfere with the binding affinity and eventual cellular internalization efficiency of the antibody component (Lee and Tannock, 2010). In addition, the antibiotic has its own distribution profile after cleavage. For instance, hydrophobic drugs may be able to interact also with non-targeted membranes, while hydrophilic drugs tend to limit their action to the antigen-expressing cell (Ferri et al., 2016). The percentage of drug that binds circulating proteins, such as albumin, also influences the drug distribution (Ascenzi et al., 2014).

Finally, AACs are cleared from the organism. The metabolism and elimination of AACs usually differs relative to the isolated (unconjugated) antibiotics. Traditional drugs are often metabolized in the liver into more polar and less active metabolites that undergo renal elimination while antibody-based systems are cleared by a complex combination of specific- and non-specific mechanisms (Ferri et al., 2016). Specific mechanisms are related to binding of antibodies to their cellular targets, which results in target-mediated clearance; or binding to FcγRs expressed on cells from the mononuclear phagocyte system (MPS). Non-specific mechanisms occur via proteolysis in a variety of tissues, such as the skin, muscle, and liver, due to macrophage uptake (Lux et al., 2013).

#### Physicochemical Properties

The physicochemical characteristics of ADCs, in general, vary significantly. In addition to the differences observed among mAbs, the drug payload plays an important part in altering physicochemical properties, which ultimately lead to deeply affected PK/PD of the ADCs (Lucas et al., 2018).

The first important physical characteristic to consider is the size of the AAC complex. Traditionally, drugs are conjugated to full IgGs (≈ 150 kDa), which possess a low clearance rate (Leelawattanachai et al., 2015). These conjugates are eliminated via cellular interaction and endocytosis, such as interaction with the MPS or proteolytic degradation (Lucas et al., 2018). Nevertheless, the conjugation to small proteins, like Fab fragments (Fabs) (≈ 50 kDa) might be of interest. As a consequence clearance is 10-fold faster than for full IgGs. Unlike full IgGs, Fabs are also eliminated in more traditional pathways, such as hepatic excretion and renal elimination (Leelawattanachai et al., 2015).

The DAR in the molecular structure of the conjugate is another critical parameter that can highly affect the PK/PD of the AAC (Hamblett et al., 2004). The optimal DAR must be assessed individually for each AAC. The conjugation of a few molecules results in a lower efficacy response; whereas the conjugation of excessive molecules might contribute to immune activation, resulting in higher clearance and increased toxicity (Sun et al., 2017). Although the best DAR is debatable, the importance of engineering homogenous AACs generates consensus among researchers, despite the difficulties it encompasses.

The modification of drug carriers is an important strategy to increase the PK of a therapeutic protein. Within antibodies, the most common is the glycosylation. It is a post-translational modification by which carbohydrates are added to specific amino acid residues (Tibbitts et al., 2016). The glycosylation sites depend on many factors, such as the cell line and pH, among others. This high heterogeneity might affect the distribution of these antibodies, which affects the efficacy and elimination (Stork et al., 2008).

Finally, another fundamental property of antibodies is the isoelectric point (pI), or the pH at which the antibody carries no net electrical charge. Traditionally, most antibodies are slightly positively charged, with a pI of 7–9 (Schoch et al., 2015). However, the manufacturing process might lead to antibodies’ heterogeneity. The differences in the surface charge affect the ADME of the AAC. Cationization, for instance, has been associated with lower absorption rates, higher clearance rates, and higher tissue accumulation. In addition, the binding affinity, extravasation, and receptor-mediated endocytosis are also affected. Anionization does not seems to affect the PK of the antibody significantly (Boswell et al., 2010).

The production of a homogeneous AAC formulation is challenging. The manufacturing parameters must be optimized and tightly controlled to ensure homogenous products. The absence of homogeneity might compromise the efficacy and the safety of an AAC with detrimental effects on patients.

### Antibody-Antibiotic Conjugates Characterization

The characterization of pharmaceutical products is crucial to ensure that the synthesis of the product follows the established guidelines and to be sure of the safety and efficacy of the product when used in patients. All components of AAC (i.e., antibody, linker and antibiotic) have different contributions to the overall physicochemical properties of the complex, conferring to each AAC its own fingerptint (Neupane and Bergquist, 2017; Kommineni et al., 2020).

Three main characteristics must be assessed for each AAC. First, the average number of molecules conjugated to an antibody in an AAC, i.e., the DAR, is particularly important. This characteristic determines the efficacy and PK property of the AAC (Dan et al., 2018). Secondly, the site of conjugation is also an important parameter as it is directly correlated to the stability and PK of the AAC (Beck et al., 2013; Strop et al., 2013). Finally, the propensity to aggregate is crucial to determine the stability of the AAC.

Several techniques, such as spectroscopic, chromatographic, and mass spectrometry (MS), have been used to characterize the AAC. Spectroscopic techniques, such as the UV-Vis absorption spectroscopy, are the easiest methods to determine DAR without extensive sample preparation. The principle of this technique is based on the specific absorption wavelength maxima (λmax) of each component (Chen, 2013). mAbs typically show λmax of 280 nm due to aromatic amino acid residues, whereas the λmax for different antibiotics varies. For successful characterization, these two λmax should be well separated. Despite its clear advantages, when the antibiotic or the linker are labile to UV radiation, this methodology cannot be applied (Petrović et al., 2005).

In the pharmaceutical industry, chromatographic methodologies have also been extensively used. A major improvement of these techniques is their versatility allowing the characterization of several physicochemical parameters (Petrović et al., 2005).

#### Hydrophobic Interaction Chromatography

The average DAR and DAR distribution can be determined by Hydrophobic Interaction Chromatography (HIC), which exploits the increased hydrophobicity of AACs with the increased number of drug loads (Neupane and Bergquist, 2017). Subsequently, distinct peaks for a different number of drug load can be observed, since the more hydrophobic the AAC, the longer the retention time. A major disadvantage of HIC is related to the non-volatile mobile phases that are usually used. These buffers are not compatible with MS detection. Therefore, standard HIC only allows the use of spectroscopic detectors, which significantly reduces the sensitivity and specificity. Recently, to overcome this issue, researchers coupled HIC with reversed-phase liquid chromatography (RPLC). This two-dimensional chromatography allows the removal of the non-volatile salts by RPLC, thus facilitating the use of MS (Birdsall et al., 2015).

#### Reversed-Phase Liquid Chromatography

RPLC is used to determine drug load distribution, DAR, and unconjugated drug of AACs complex (Valliere-Douglass et al., 2012). In addition, it has also been applied in assessing of the stability of AACs under different storage conditions. The major variables to consider are the gradient steepness, mobile phase temperature, and mobile phase ternary composition (Fekete et al., 2017). A major advantage over HIC is the volatile nature of the mobile phase and buffers used since they allow detection through MS. Nevertheless, antibodies have a strong tendency to bind strongly to the reversed-phase column, which results in unspecific losses that must be considered (Lazar et al., 2005).

#### Size-Exclusion Chromatography

Size-Exclusion Chromatography (SEC) analysis is essentially used to detect aggregation formed in either the synthesis or the storage of the AAC (Kommineni et al., 2020). In the engineering of antibodies, aggregation represents a serious concern owing to the stimulation of immune reactions, changes in the PK properties of antibodies, like the clearance rate, and their binding specificity (Sauerborn and van Dongen, 2014). The interaction of the AAC to the column can occur by electrostatic or hydrophobic interactions. Depending on the type of interaction, the elution time and tailing are affected, which also affects the characterization of the AAC. Currently, this technique is increasing in popularity for the study of unconjugated drugs and the quantification of excipients in the pharmaceutical formulation of antibodies and AACs (He et al., 2012).

#### Mass Spectrometry

MS is a very sensitive technique that detects small differences in the mass of the AAC (Neupane and Bergquist, 2017). The antibiotic, the linker, and the antibody possess distinct masses, creating a fingerprint for each AAC. Thus, all components must be very well described for the success of MS detection. Intact mass of AACs can be determined with an accuracy of 30–100 ppm range that enables characterization of DAR, heterogeneity, and antigen-binding stoichiometries of AACs (Beck et al., 2016). Additionally, it is likely to identify the site of conjugation and domain sequence in AAC. To that end, tandem MS must be used, often after digestion to fragments with suitable enzymes. Finally, researchers can also monitor single reactions for the determination of unconjugated drugs or drug-linker residues in the pharmaceutical product.

## The Case Study of DSTA4637A

*Staphylococcus aureus* is responsible for most bacterial infections worldwide in humans and represents a significant health problem in hospitals and community settings (Diekema et al., 2001). Unfortunately, infections with *Staphylococcus aureus* have become increasingly challenging to treat due to the emergence and rapid spread of methicillin-resistant *Staphylococcus aureus* (MRSA) strains, combined with dose-limiting adverse effects with current antibiotics, such as vancomycin and nafcillin. In addition, considerable evidence suggests that this remarkable bacterial survival might be attributed to harboring and growth inside host cells (Boucher et al., 2009), where bacteria are protected from host defenses (Thwaites and Gant, 2011). Moreover, host cells presumed to protect the host, such as the phagocytes, can increase bacteria dissemination to other sites. Together, this evidence leads researchers to develop a novel and innovative strategy to eliminate both the dormant and the intracellular bacteria (Lehar et al., 2015).

**FIGURE 3:**
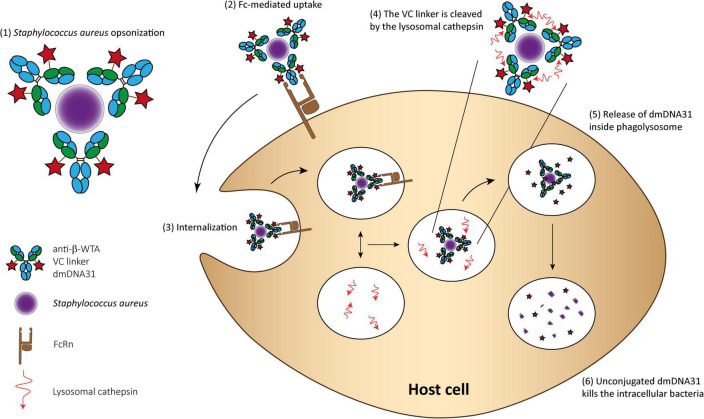
THIOMAB™ AAC mechanism of action for killing Staphylococcus aureus. (1) The AAC binds Staphylococcus aureus bacteria; (2) The Fc domain of the monoclonal antibody is recognized by the FcRn on the surface of professional phagocytes or other host cells, such as epithelial cells; (3) The complex is internalized; (4) Fusion between the phagosome and lysosome and cleavage of the VC linker; (5) The active dmDNA31 is released attacking the intracellular bacteria; and (6) Unconjugated dmDNA31 eliminates the intracellular bacteria.

An AAC THIOMAB™ named DSTA4637A was developed to eliminate intracellular *Staphylococcus aureus* and is currently under clinical trials (Lehar et al., 2015; Zhou et al., 2016; Peck et al., 2019). This AAC consists of (1) a monoclonal human immunoglobulin (IgG1) antibody that specifically binds to wall teichoic acids of *Staphylococcus aureus*; (2) a novel antibiotic 4-dimethylaminopipepidino-hydroxybenzoxazino rifamycin (dmDNA31), a rifampin-class antibiotic with an *in vitro* minimum inhibitory concentration (MIC) < 10 nM toward MRSA; and (3) a protease cleavable valine-citrulline linker that allows antibiotic release inside phagosomes. The proposed mechanism of action involves the binding of the AAC to *Staphylococcus aureus* surface antigen resulting in the opsonization of the bacteria. Then, inside host cells, host proteases in the phagolysosome, like cathepsins, cleave the linker and the antibody is released in its active form. In addition, it is hypothesized that when bacteria are released from the intracellular reservoirs, the prolonged presence of the AAC, due to the high circulation time of such molecules, immediately “tag” these bacteria for elimination (Figure 3; Mariathasan and Tan, 2017).

DSTA4637A, demonstrated potent elimination of *Staphylococcus aureus in vitro* and *in vivo* (Lehar et al., 2015; Zhou et al., 2016). Moreover, this AAC was considered superior to vancomycin for treating bacteremia. In addition, for characterization of efficacy, researchers also investigated the PK/PD of DSTA4637A in infected and non-infected mouse models (Zhou et al., 2016). Its PK profile is bi-phasic, characterized by a short distribution phase and a prolonged elimination phase as expected for monoclonal antibody-based therapeutics. The conjugation to the antibody also significantly improved the PK profile of the antibiotic. In addition, the half-life of dmDNA31 was extended from 3 to 4 h to approximately 4 days. Consequently, more antibiotic accumulates in cellular regions where *Staphylococcus aureus* is present, improving its efficacy and reducing the therapeutic dose, resulting in less adverse events. Interestingly, the administration of DSTA4637A demonstrates a substantially reduced bacterial load in the heart, kidney, and bones on 7 and 14 days-post dosing.

Next, DSTA4637S, the clinical formulation of DSTA4637A, was tested in a Phase 1 study to investigate the safety, tolerability, and PK in thirty healthy volunteers (Peck et al., 2019). During the 85 days follow-up, no subject withdrew from the study, and no serious or severe adverse events occurred. PK of plasma DTSA4637S conjugate and serum DSTA4637S total antibody were dose-proportional. Very low levels of unconjugated dmDNA31 were observed, and no anti-drug antibodies (ADAs) were detected. Consequently, these results support the future development of this THIOMAB™ AAC as a novel therapeutic for *Staphylococcus aureus* infections.

Using antibiotics as payloads in the development off AACs is a valuable strategy. Nevertheless, other therapeutic molecules can be conjugated to antibodies, such as, AMPs or enzymes that can create effective ADCs for infectious diseases (Cavaco et al., 2018).

## What Does the Future Holds for Antibody-Drug Conjugates?

### The Use of Peptidomimetics

The use of antibiotics has significantly improved the treatment of bacterial infections. However, the increase of resistant pathogens has leveraged the study of innovative strategies (Mood et al., 2021). As a result, the use of peptides emerged as a promising alternative (Rima et al., 2021). Peptides are a broad group of molecules with different physicochemical properties and therapeutic indications. The use of antimicrobial peptides (AMPs) concerning bacterial infections (Mahlapuu et al., 2016; Lei et al., 2019; Datta and Roy, 2021) is a matter of intense research. They possess numerous important properties, such as specificity, potency, low toxicity, biological diversity, and unique mechanisms of action (bacterial membrane and/or cytoplasmic), differing from conventional antibiotics, which can impact the new era of antimicrobials due to decreased bacterial resistance (Zhang et al., 2021).

There are several AMPs in preclinical and clinical development (Huan et al., 2020; Datta and Roy, 2021). Unfortunately, the immense flood of AMPs under investigation do not translate into a high number of approved AMPs. Overall, the main disadvantage of peptides regarding their clinical use is related to their physiological stability (Cavaco et al., 2021a). Peptidomimetics have been investigated to overcome this limitation, as different chemical and physical alterations can be applied to increase proteolytic stability, enhance bioavailability, and improve PK (Qvit et al., 2017; Lenci and Trabocchi, 2020; Del Gatto et al., 2021). These peptides are obtained *via* chemical synthesis, which is a simple, fast, and high yield methodology (Cavaco et al., 2018). Nonetheless, the generation of peptidomimetics might not be sufficient to further improve peptides’ applicability. Thus, as for drugs and antibiotics, peptides can also be conjugated to antibodies to improve their physicochemical properties, creating peptibodies (Cavaco et al., 2018). The engineered drug-delivery system will take advantage of the selectivity and long t_1/2_ of antibodies.

In the literature, so far, there are no promising examples of peptibodies targeting bacterial infections. The use of recombinant DNA technology is the most effective strategy to generate peptibodies (Wu et al., 2016). However, since the generation of peptidomimetics relies on the chemical synthesis of peptides to incorporate, for instance, unnatural amino acids, D-amino acids or chemical modifications, the conjugation to antibodies must be performed *via* chemical (copper-free click chemistry, sortase-mediated protein ligation, streamlined-expressed protein ligation) or enzymatic reactions (Fanny et al., 2007). Overall, the main disadvantage of such techniques is the use of high temperature and/or low pH, which might affect the physicochemical properties of peptides. This feature might be impacting the generation of peptibodies toward bacterial infections. Nevertheless, the use of streamlined-expressed protein ligation, which relies on the use of ultrafast split-inteins for protein α-thioester generation, has been proposed as a promising strategy to overcome these limitations (Shah et al., 2012; Vila-Perelló et al., 2013). It presents high selectivity, lower time reactions, high yields, and high purity. So far, there are some examples reporting the success of streamlined-expressed protein ligation (Shimamoto et al., 2012; Cavaco et al., 2018, 2021b; Frutos et al., 2018); however, it is expected an increase use of such technique to conjugate peptides/peptidomimetics to antibodies, which might lead to better therapeutic options in order to eliminate bacterial infections.

### The Use for Non-bacterial Infectious Diseases

Bacterial infections are a major threat to public health since the resistance to antibiotics is rising exponentially. Nevertheless, other infectious diseases can affect both humans and animals with a substantial impact on human health and the economy (Smith et al., 2019).

The infection with African trypanosomes is a good example of an infection that can cause disease in humans and livestock. The current treatments are not always effective and often present severe side effects since they require multiple administrations over long periods (Barrett, 2018). Without intervention, the infection persists due to the antigen variation of the variant surface glycoprotein (VSG) on the trypanosome plasma membrane (Nielsen et al., 2014).

An early attempt to exploit VSG as targets for therapeutic delivery lead to the development of two distinct ADCs. The first one comprises the conjugation of chlorambucil to polyclonal IgGs purified from chronically infected rabbits (Carvalhaes et al., 1998). While the results were promising, some adverse events limited the applicability of such a molecule. More recently, researchers explored the wide distribution of VSG on the plasma membrane of trypanosomes (Stijlemans et al., 2004, 2017). They engineered several nanobodies conjugated to effective drugs that could specifically bind and kill trypanosomes. Unfortunately, due to VSGs’ high variability, parasites quickly become resistant to such treatments.

Nevertheless, some features are conserved during the antigen variation of the parasite. For example, incorporating receptors for host nutrient macromolecules, such as the haptoglobin-hemoglobin receptor (HpHbR), is a crucial component of parasite survival (Vanhollebeke et al., 2008). The HpHbR is involved in haem acquisition through the endocytosis of host haptoglobin-hemoglobin. Since it remains unmodified during the parasite antigen variation, it represents an attractive target for target-selective therapies. In an attempt to explore this pathway, researchers developed a recombinant human anti-trypanosome-HpHbR conjugated to a pyrrolobenzodiazepine (PBD) toxin (MacGregor et al., 2019). The antibody-PBD conjugate was effective at killing trypanosomes *in vitro* at picomolar concentrations. In addition, a single dose of the ADCs resulted in a long-term cure in the standard mouse model of trypanosome infection.

Another non-bacterial and non-parasite application might be viral infections. The recent viral outbreaks demonstrated that our therapeutical arsenal is not sufficiently vast and efficient to control a pandemic quickly. Thus, due to the importance that antibodies encompass in controlling naturally occurring viral infections, researchers might be tempted to conjugate antiviral agents to antibodies targeting viral particles. This new strategy will face some problems already described for ADCs and AACs. However, the decades of research in this area will guide them in developing efficient conjugates.

## Author Contributions

All authors listed have made a substantial, direct, and intellectual contribution to the work, and approved it for publication.

## Conflict of Interest

The authors declare that the research was conducted in the absence of any commercial or financial relationships that could be construed as a potential conflict of interest.

## Publisher’s Note

All claims expressed in this article are solely those of the authors and do not necessarily represent those of their affiliated organizations, or those of the publisher, the editors and the reviewers. Any product that may be evaluated in this article, or claim that may be made by its manufacturer, is not guaranteed or endorsed by the publisher.
